# Genome editing of CCR5 by AsCpf1 renders CD4^+^T cells resistance to HIV-1 infection

**DOI:** 10.1186/s13578-020-00444-w

**Published:** 2020-07-08

**Authors:** Zhepeng Liu, Jin Liang, Shuliang Chen, Kewu Wang, Xianhao Liu, Beibei Liu, Yang Xia, Mingxiong Guo, Xiaoshi Zhang, Guihong Sun, Geng Tian

**Affiliations:** 1 Department of Biotherapy Research Center, Sun Yat-sen University Cancer Center, 651 Dongfeng East Road, Guangzhou, Guangdong 510060 People’s Republic of China; 2 Department of Oncology, The First Affiliated Hospital of Shenzhen University, The Second People’s Hospital of Shenzhen, 3002 Sungang West Road, Shenzhen, 518035 People’s Republic of China; 3 School of Basic Medical Sciences, Wuhan University, Wuhan, 430071 People’s Republic of China; 4 Department of Oncology, The Second People’s Hospital of Wuhu, Wuhu, 242401 People’s Republic of China; 5 College of Life Sciences, Wuhan University, Wuhan, 430071 People’s Republic of China

**Keywords:** CCR5, HIV-1, CRISPR/AsCpf1, HIV-1 resistance, Selective advantage

## Abstract

**Background:**

The chemokine receptor CCR5 is one of the co-receptor of HIV-1 infection. People with homozygous *CCR5Δ32* deletion resist HIV-1 infection, which makes the *CCR5* an important target for HIV-1 gene therapy. Although the CRISPR/Cas9 has ever been used for HIV-1 study, the newly developed CRISPR/AsCpf1 has never been utilized in HIV-1 co-receptor disruption. The CRISPR/Cpf1 system shows many advantages over CRISPR/Cas9, such as lower off-target, small size of nuclease, easy sgRNA design for multiplex gene editing, etc. Therefore, the CRISPR/Cpf1 mediated gene editing will confer a more specific and safe strategy in HIV-1 co-receptor disruption.

**Results:**

Here, we demonstrated that CRISPR/AsCpf1 could ablate the main co-receptor of HIV-1 infection-*CCR5* efficiently with two screened sgRNAs via different delivery strategies (lentivirus, adenovirus). The edited cells resisted R5-tropic HIV-1 infection but not X4-tropic HIV-1 infection compared with the control group in different cell types of HIV-1 study (TZM.bl, SupT1-R5, Primary CD4^+^T cells). Meanwhile, the edited cells exhibited selective advantage over unedited cells while under the pressure of R5-tropic HIV-1. Furthermore, we clarified that the predicted off-target sites of selected sgRNAs were very limited, which is much less than regular using sgRNAs for CRISPR/Cas9, and no evident off-target was observed. We also showed that the disruption of *CCR5* by CRISPR/AsCpf1 took no effects on cell proliferation and apoptosis.

**Conclusions:**

Our study provides a basis for a possible application of *CCR5*-targeting gene editing by CRISPR/AsCpf1 with high specific sgRNAs against HIV-1 infection.

## Background

Acquired immunodeficiency syndrome (AIDS), caused by HIV-1 (human immune deficiency virus type 1) infection has threatened the health of people all over the world. According to the statistic data from World Health Organization in 2018, 37.9 million people are living with HIV globally, and 23.3 million people (62%) were receiving highly active antiretroviral therapy (HAART) (https://www.who.int/hiv/data/en/).

However, HAART can not eradicate the HIV-1 virus, for the integration of HIV-1 into host genome. Moreover, the HAART has many limitations, such as long term drug taking, high cost and side effects (hepatic lesion, cardiovascular diseases, etc.) [1–3]. Therefore, it is necessary and inevitable to look for alternative and more effective therapies to cure HIV-1 infection.

HIV-1 infection of host cells is a multi-step process, the virus entry into cells begin with viral envelop protein gp120 binding to CD4 and one of the co-receptors CCR5 or CXCR4 on cell surface, the co-receptor CCR5 or CXCR4 is required for different HIV-1 strains, with CCR5 for CCR5(R5)-tropic strain and CXCR4 for CXCR4(X4)-tropic strains [4, 5]. The genomic RNA of the virus will be converted to double-strand DNA after virus entering cells by membrane infusion. The viral DNA will integrate into target cell genome to be the provirus, the provirus can be transcribed as viral RNAs, which will be packaged to be a new virus particle. Previous reports have clarified that individuals with homozygous deletion of co-receptor CCR5 (*CCR5*∆*32*) are resistant to HIV-1 infection [6–8]. Meanwhile, clinical reports of two patients (*Berlin Patient, London Patient*) with HIV-1 infection and leukemia received HLA-matched and homozygous *CCR5*∆*32* bone marrow transplant have been proved that they got clinic-defined cure with undetectable HIV-1 [9–11]. Therefore, the co-receptor CCR5 has been a reasonable target for gene editing against HIV-1 infection.

Over last decades, several genome editing tools have been developed and utilized for diseases study and cure, such as zinc finger nuclease (ZFN), transcription activator like effector nucleases (TALEN), which have been proven to be efficient *CCR5* gene editing tools [12–16]. In the year of 2013, Feng Zhang and George Church et al. developed Clustered regularly interspaced short palindromic repeats (CRISPR) and CRISPR associated nuclease 9 (CRISPR-Cas9) gene modification technique, which has resulted revolution in gene editing [17, 18]. CRISPR/Cas9 technology takes several advantages over ZFN and TALEN, such as easy design, high efficiency, etc. Hence, the technology has also been applied to mediate HIV-1 co-receptor editing [19–22]. Li et al. has reported that they have disrupted *CCR5* in different CD4^+^T cells, which has protected the edited cells from HIV-1 (R5-strains) infection. Meanwhile, analyzing the most effective 3 sgRNAs and their corresponding 15 potential off-target sites revealed that no significant editing efficacy in these sites [23]. For the co-receptor CXCR4, Hou et al. has proven that the disruption of *CXCR4* by CRISPR/SpCas9 in genome level confers the edited cells resistant to HIV-1(X4-strains) infection and no obvious effects on off-target and proliferation, Wang et al. has verified the phenomenon with *CXCR4* modification by CRISPR/SaCas9 [20, 24]. Some works about simultaneous editing of HIV-1 co-receptor CCR5 and CXCR4 by CRISPR/Cas9 have also been reported, Yu et al. and our previous work have confirmed that the two genes could be disrupted simultaneously in genome level and the edited cells could resist R5-tropic strain and X4-tropic strain concurrently with survival advantage over unedited cells under mixed HIV-1 infection pressure [25, 26]. Recently, Xu et al. have reported they have utilized CRISPR/Cas9 to edit *CCR5* gene in HSPCS and transplant the cells to a acute lymphoblastic leukemia (ALL) and HIV-1 bearing 27 years old male in China. The ALL was complete remission, and donor cells carrying the disrupted *CCR5* persisted for more than 19 months without related adverse events. The percentage of edited cells with *CCR5* ablation increased by a small degree during a short of anti retroviral therapy pause [27].

The CRISPR/Cpf1 technology was first reported in 2016 and the nuclease Cpf1 has three different origins, namely *Acidaminococcus* (AsCpf1), *Lachnospiraceae bacterium*(LbCpf1), *Francisella novicida*(FnCpf1) [28, 29]. Compared with the well-known CRISPR/spCas9, CRISPR/Cpf1 displays many difference, such as the protospacer-adjacent motif (PAM) sequence of Cpf1 is 5′-TTTN-3′ but not 5′-NGG-3′ of spCas9, which makes the sgRNAs more specific. The size of nuclease Cpf1 is much smaller than SpCas9, indicating the feasibility of the expressing the system in adenovirus (Adv) or adeno-associated virus (AAV). The sgRNA using by Cpf1 to bind target sequence consists of CRSIPR RNA (crRNA) only without trans-activating CRISPR RNA (tracRNA) in spCas9, for the nuclease can process the maturity of crRNA by itself, which means the multiple- target design will be much more easier than spCas9 [28, 30]. The CRISPR/Cpf1 technology, like CRISPR/spCas9, has also been utilized in biological and medical study. For example, Dai et al. has edited *TRAC* and *PDCD1* gene in the generation of modular CAR-T cells [31]. Wang et al. has successfully adopted the technology to modify plants phenotype associated genes [32]. However, no report has been published about HIV-1 co-receptor CCR5 or CXCR4 editing with CRISPR/Cpf1.

In this study, we screened and identified two CRISPR/AsCpf1 using sgRNAs with high specificity to target *CCR5*. With different CD4^+^T cells (Tzm.bl, SupT1-R5, primary CD4^+^T cells), we demonstrated that CRISPR/AsCpf1 could ablate *CCR5* gene efficiently in all cell types and confer the edited cells resistant to R5-tropic strain but not X4-tropic strain infection. Meanwhile, the edited cells show survival advantage over unedited cells under R5-tropic strain infection pressure.

## Results

### Lenti-AsCpf1 mediated *CCR5* disruption in adherent CD4^+^ TZM.bl cell line

**Fig. 1:**
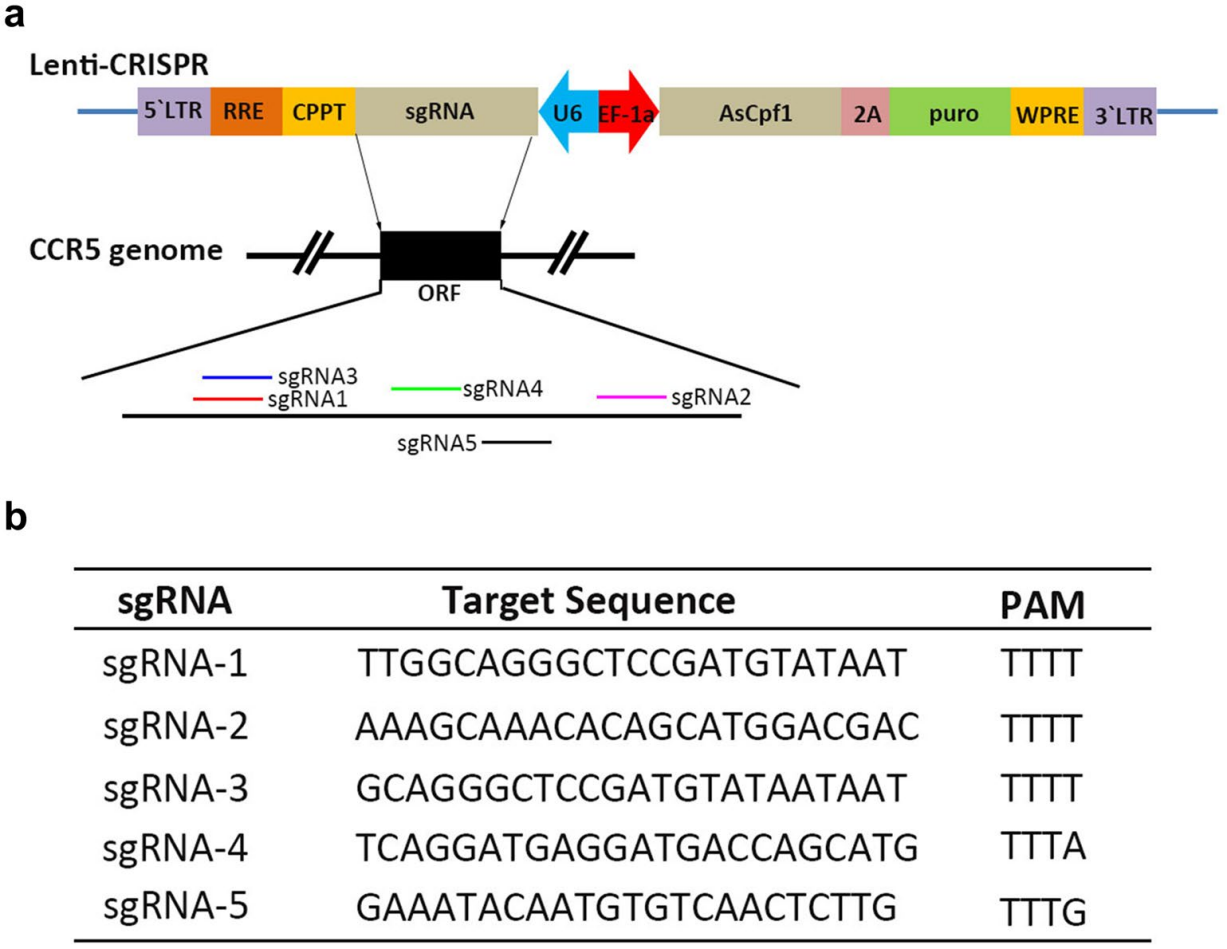
Schematic diagram of CRISPR/AsCpf1 mediating *CCR5* disruption. **a** Diagram of all the *CCR5* sites targeted by CRISPR/Ascpf1 sgRNAs. **b** sgRNAs used in the identification of the most efficient sgRNAs screen in *CCR5* disruption

**Fig. 2:**
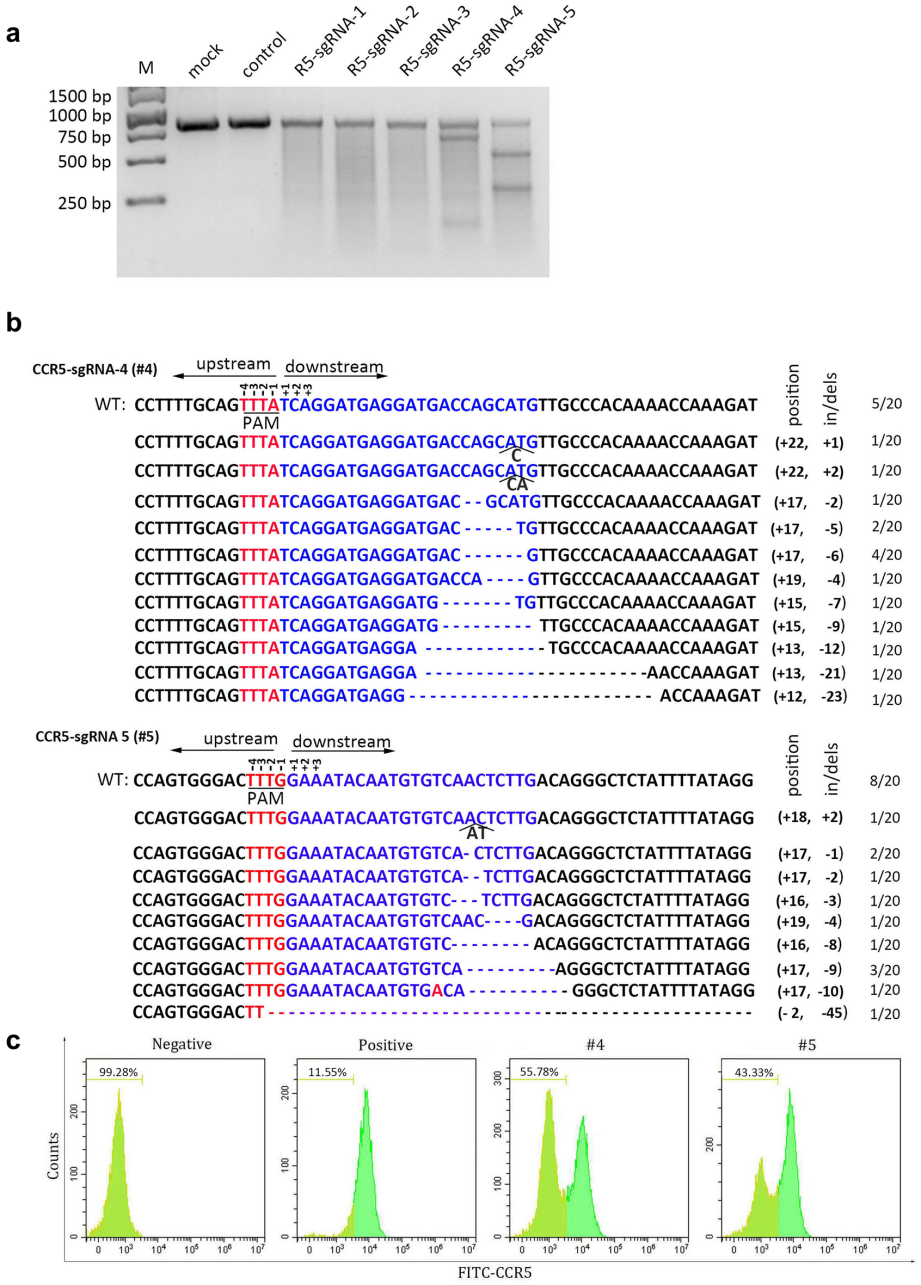
Lenti-CRISPR/AsCpf1 mediated the disruption of *CCR5* in TZM.bl cells. **a** 5 sgRNAs for *CCR5* editing by CRISPR/AsCpf1 were screened and identified by T7 endonuclease 1 (T7E1). **b** The amplicons of #4 and #5 were subjected to DNA sequencing by ligating with PGEM-T easy vector. **c** the protein level of CCR5 on cell surface were analyzed by flow cytometry at the third day post-transfection

To silence CCR5 expression, we designed 5 sgRNAs targeting different sites within *CCR5* exon (Fig. 1a, b), and cloned the sgRNAs to lenti-AsCpf1 backbone after U6 promoter. The constructed plasmids and control plasmid were transfected into TZM.bl cell lines, an adherent cell line with high expression of CD4 and HIV-1 co-receptor, CCR5 and CXCR4 on cell surface. The transfected cells were added and incubated with 1 µg/ml puromycin for 24 h after the first day, the treated cells were collected and analyzed with T7E1 assay which cleaves DNA at distorted duplexes caused by mismatches. The related primers are listed in Additional file 1: Table S1. The result showed that the designed #4, #5 could cleave the 828 bp *CCR5* PCR amplicon, while the blank and control showed no obvious cleaved bands (Fig. 2a), which indicated the lenti-AsCpf1 could mediate *CCR5* ablation in TZM.bl cell line with sgRNA #4 and #5 efficiently. DNA sequencing has also been performed to analyze the in/dels (insert/deletion) efficacy of each sgRNA, the results showed the #4 leaded 15 out of 20 editing (75.0%) and #5 induced editing ratio was 12/20 (60.0%) (Fig. 2b), which implied that the #4 might be more efficiently in *CCR5* disruption than #5. Furthermore, to identify if the *CCR5* genome level ablation mediated CCR5 protein expression regulation on cell surface, flow cytometry was performed to measure the CCR5 protein on cell surface (Fig. 2c). The results displayed that #4, #5, compared with positive control, exhibited 44.23% and 31.78% CCR5 protein down-regulation respectively. To investigate if the effect of *CCR5* editing with AsCpf1 or spCas9 was different, two CRISPR/spCas9-sgRNA (#1, #2) were constructed and compared with CRISPR/AsCpf1-#4, among which the sgRNAs overlap each other (Additional file 2: Fig S1). According to the results, the *CCR5* editing ratio of Cas9-#1, #2 ranged from 60.0% to 70.0%, which showed no difference comparing with AsCpf1-#4 (60.0%). Besides, the top 3 ranking candidates of all predicted off-target sites for Cas9-#1, #2 have been analyzed with DNA sequencing and T7E1, and the results revealed that the selected off-target sites for analysis exhibited no disruption. The results indicated that both AsCpf1 and spCas9, with target sequence overlap each other in this study, could ablate *CCR5* in genome level efficiently.

### *CCR5* ablation could be induced by AsCpf1-sgRNA packaged lentivirus in SupT1-R5 cells

**Fig. 3:**
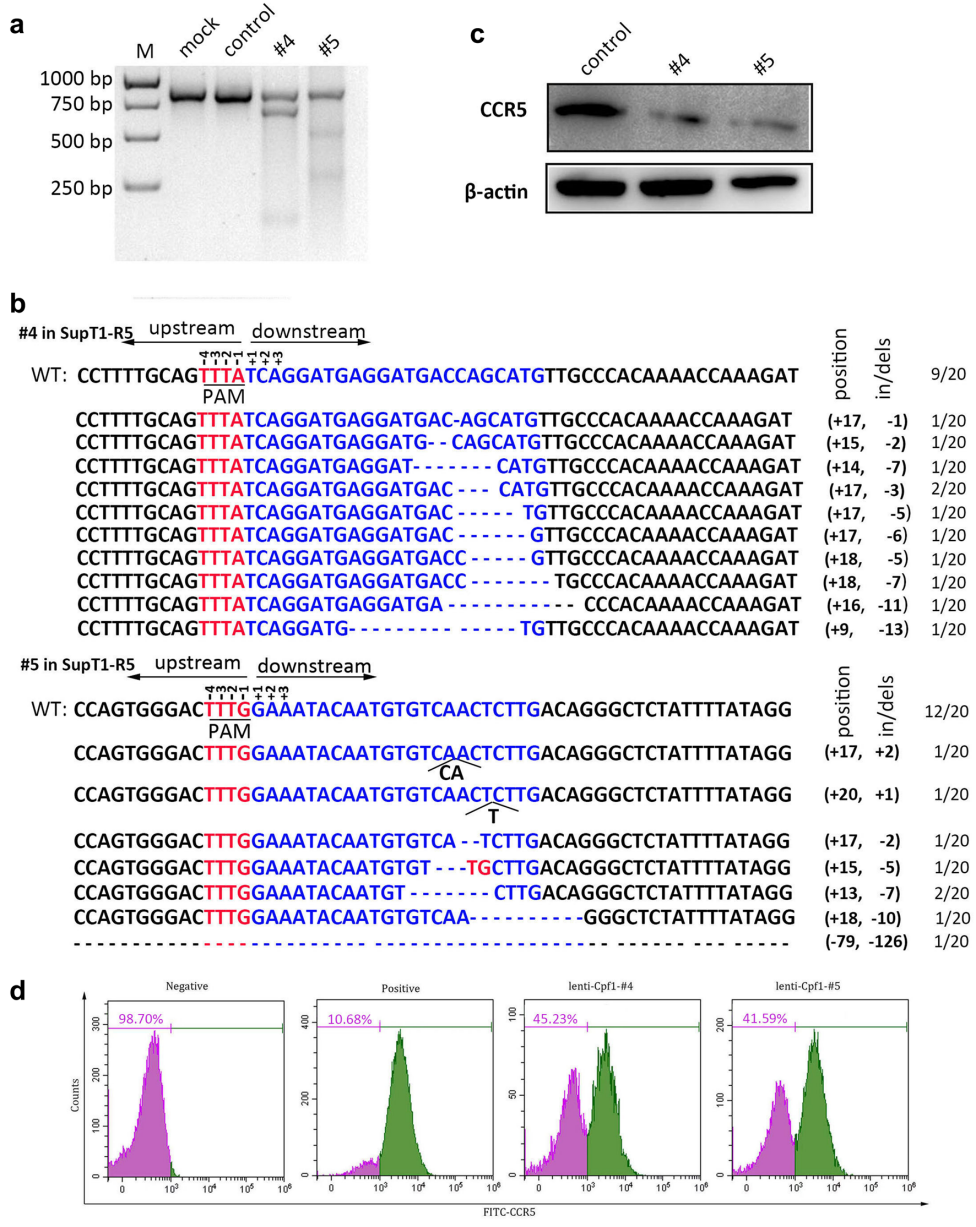
The CRISPR/AsCpf1-#4/#5 expressing lentivirus induced genome editing of *CCR5* in SupT1-R5 cells. **a** 300 ng PCR products of #4 and #5 or control were analyzed by T7E1 assay after lenti-CRISPR/AsCpf1 mediated CCR5 disruption at day 3. **b** The amplicons of #4 and #5 were treated with poly A adding kit, and the products were further ligated with PGEM-T easy vector and analyzed with DNA sequencing. **c** CCR5 protein level of the edited SupT1-R5 cells were detected at day 7 with 10 µg load after packaged lentivirus transduction. **d** The CCR5 expression on cell surface were assessed with FACS

The major target cell infected by HIV-1 in human body is primary CD4^+^T cells, a suspension cell type. Thus, we further tested if the CRISPR-AsCpf1 could work in SupT1-R5 cells, which is a common model of suspension cells in HIV-1 study. The AsCpf1-sgRNA -#4/#5 and control were packaged into lentivirus respectively, and then the virus were used to transduce the SupT1-R5 cells. The treated cells were collected and performed with T7E1 assay after 3 days post transduction. Obvious *CCR5* cleaved fragments could be observed of #4 or #5, but the control or blank showed no disruption (Fig. 3a). Consistently, DNA sequencing was performed for the PCR product of *CCR5* (Fig. 3b), the result showed 11/20 of #4 (55%) and 8/20 of #5 (40%) were processed with indels after the corresponding lentivirus transduction, which indicated the high efficacy of lenti-CRISPR/AsCpf1-#4/#5 mediated *CCR5* disruption. In a parallel assay, 7 days after transduction, the whole protein was collected and analyzed with immunoblotting to detect the CCR5 protein change in each treated cells (Fig. 3c), the result showed that the CCR5 protein levels were markedly down-regulated with transduction of #4 or #5 lentivirus compared to control. In addition, the flow cytometry was also performed to analyze the CCR5 expression on cell surface. The results showed that the CCR5 protein expression level were down-regulated by 34.55% in #4 and 30.91% in #5 compared with control (Fig. 3d). The same results could be observed with another suspension CD4^+^ T cell-Jurkat T cell line (data not shown).

### The edited CD4^+^T cells inhibits HIV-1 infection

**Fig. 4:**
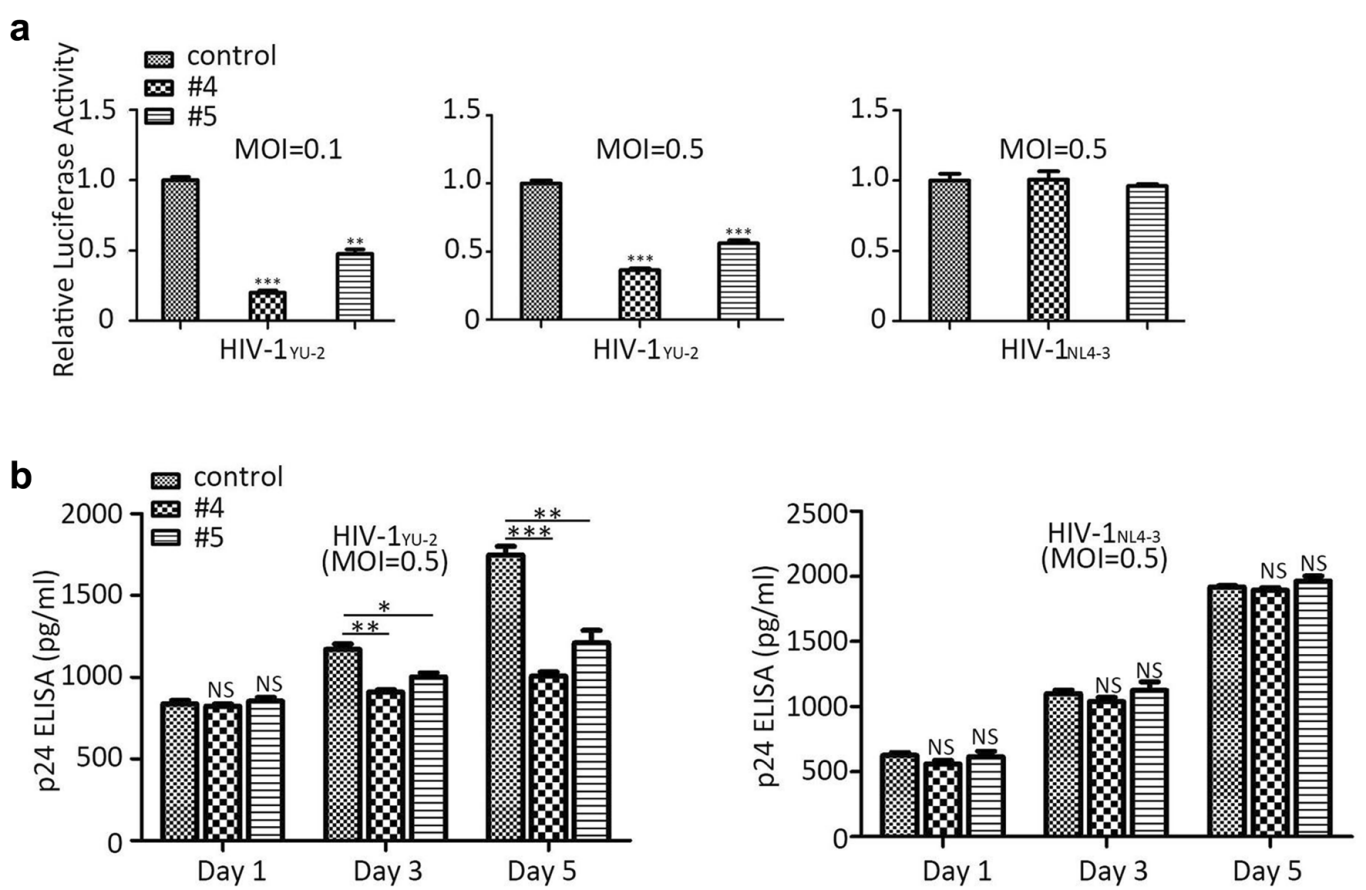
The *CCR5* edited TZM.bl and SupT1-R5 cells were protected from R5-tropic HIV-1infection. **a** Luciferase report assay to identify the HIV-1 infection level in TZM.bl. The edited cells were infected with R5-tropic HIV-1_YU-2_ or X4-tropic HIV-1_NL4-3_ at MOI = 0.5 or 0.1, cells were collected and lysed with 100 μl lysis buffer for luciferase activity detection at day 5. **b** The *CCR5* modified SupT1-R5 cells were challenged with R5-tropic HIV-1_YU-2_ or X4-tropic HIV-1_NL4-3_ at MOI = 0.5. The data shown were the mean ± SD of three independent experiments. *P < 0.05; **P < 0.01;***P < 0.001; NS, not significant; Statistical analysis determined using unpaired t-test

To test whether the *CCR5* modification would render the cells resistant to HIV-1 infection, two HIV-1 variants (HIV-1_YU-2_, HIV-1_NL4-3_) were used to infect the edited cell types above and the corresponding control cells. As shown in (Fig. 4a) at day 5 post-infection, the modified TZM.bl cells showed significant R5-tropic variant (HIV-1_YU-2_) resistance at MOI = 0.1 or 0.5 compared with control. However, the edited TZM.bl cells exhibited no difference while X4-tropic variant (HIV-1_NL4-3_) was used at MOI = 0.5. The same experimental phenomenon could be observed with the suspension cell type-SupT1-R5 from day 1 to day 5 post-transduction (Fig. 4b). The results indicated that the disruption of *CCR5* mediated by lenti-CRISPR-#4/#5 in TZM.bl and SupT1-R5 cells, rendered the cells resistant to R5-tropic HIV-1 infection but not X4-tropic infection. In addition, as the CXCR4 was another co-receptor of HIV-1 infection, we have also screened and edited *CXCR4* in genome successfully with CRISPR/AsCpf1 in TZM.bl cells, and the co-receptors *CCR5* and *CXCR4* have been ablated simultaneously by co-transfection the corresponding plasmids (AsCpf1-CCR5-#4 and AsCpf1-CXCR4-#2) into TZM.bl cells. Interestingly, the *CCR5* and *CXCR4* edited cells could resist HIV-1_YU-2_ and HIV-1_NL4-3_ infection concurrently, while the single co-receptor edited groups showed no difference when compared with control (Additional file 3: Fig S2).

### The edited SupT1-R5 cells gain a selective advantage over unedited cells

**Fig. 5:**
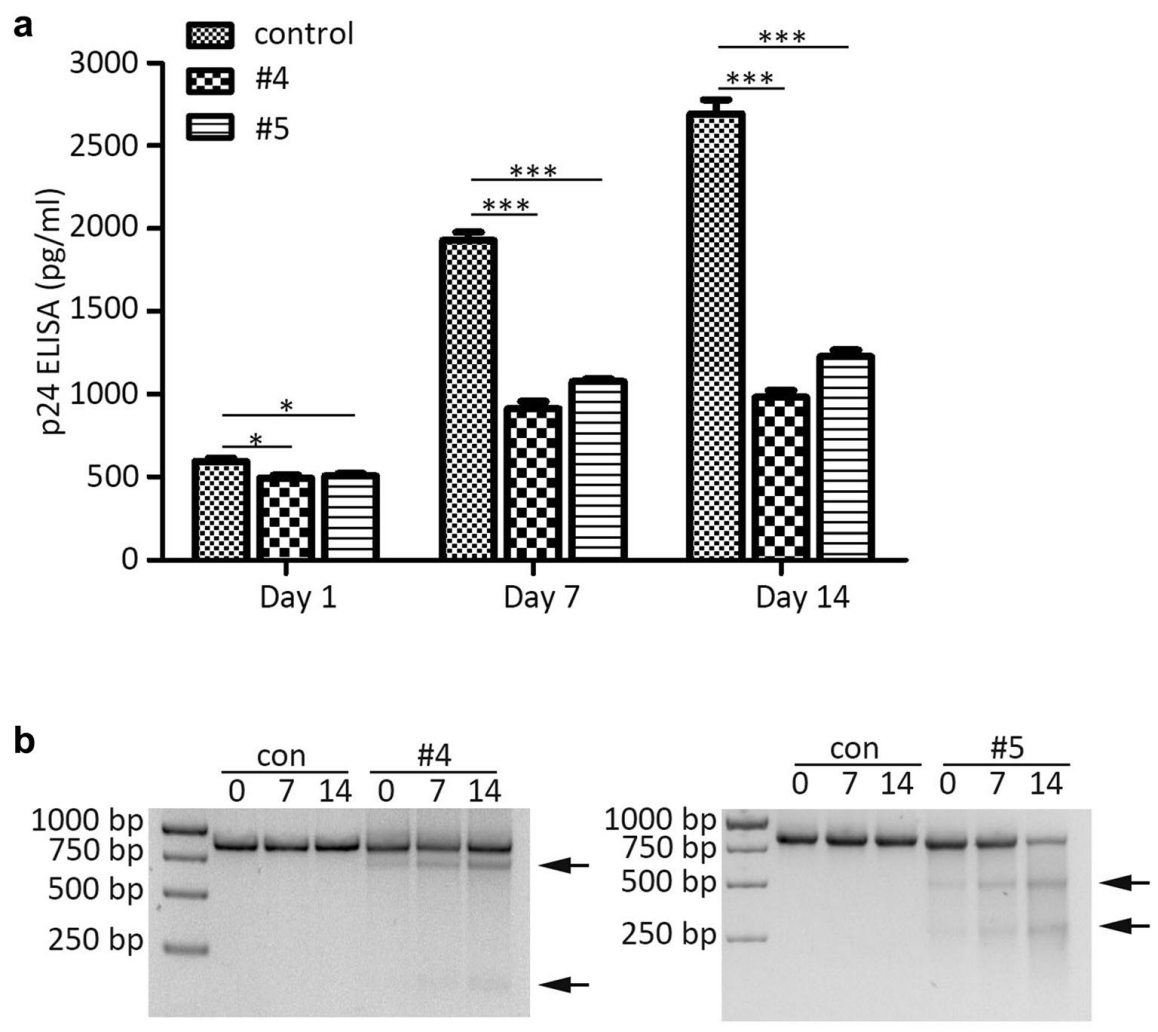
The CCR5 ablated SupT1-R5 cells gain selective advantage over unmodified cells. **a** The SupT1-R5 cells were transduced with lenti-CRISPR/AsCpf1-#4/#5 or control lentivirus at MOI = 30 for 4 days, the treated cells were then infected with R5-tropic HIV-1_YU-2_ at MOI = 0.5. The HIV-1 titer in supernatant of each cells at day 1, 7, 14 post-infection were determined by p24 gag ELISA kit. **b** The cleavage of CCR5 fragments at day 0, 7, 14 post-infection were analyzed by T7E1. The data shown were the mean ± SD of three independent experiments. *P < 0.05; **P < 0.01;***P < 0.001; Statistical analysis determined using unpaired t-test

In order to rule out the effect of multiple rounds of infection in unedited cells, we performed a selective advantage assay by exposing the treated SupT1-R5 cells to R5-tropic HIV-1_YU-2_ and prolonging the culture time for 14 days. The culture supernatant (day 1, day 7, day 14) of each group were collected and analyzed with HIV-1 gag p24 measurement (Fig. 5a). From the result, the unmodified control showed a continuous increase of the HIV-1 titer, but the modified groups exhibited a mild rise. Meanwhile, T7E1 assay was performed to evaluate the *CCR5* ablation of edited cells and control cells at day 0, day 7 and day 14 (Fig. 5b), the result demonstrated that the edited groups (#4 and #5) displayed an obvious increase of lower migrating bands corresponding to cleavage products, but the control group showed no editing persistently. The results suggested that the #4, #5 modified SupT1-R5 cells were enriched during HIV-1_YU-2_ infection, and the cells were conferred with increased HIV-1 resistance ability as the infection processing. Thus, the conclusion could be drawn that the #4 or #5 edited SupT1-R5 was resistant to HIV-1_YU-2_ infection and exhibited a survival advantage over unedited control cells.

### *CCR5* disruption protects primary CD4^+^T cells from HIV-1 infection by Adv-CRISPR/AsCpf1

**Fig. 6:**
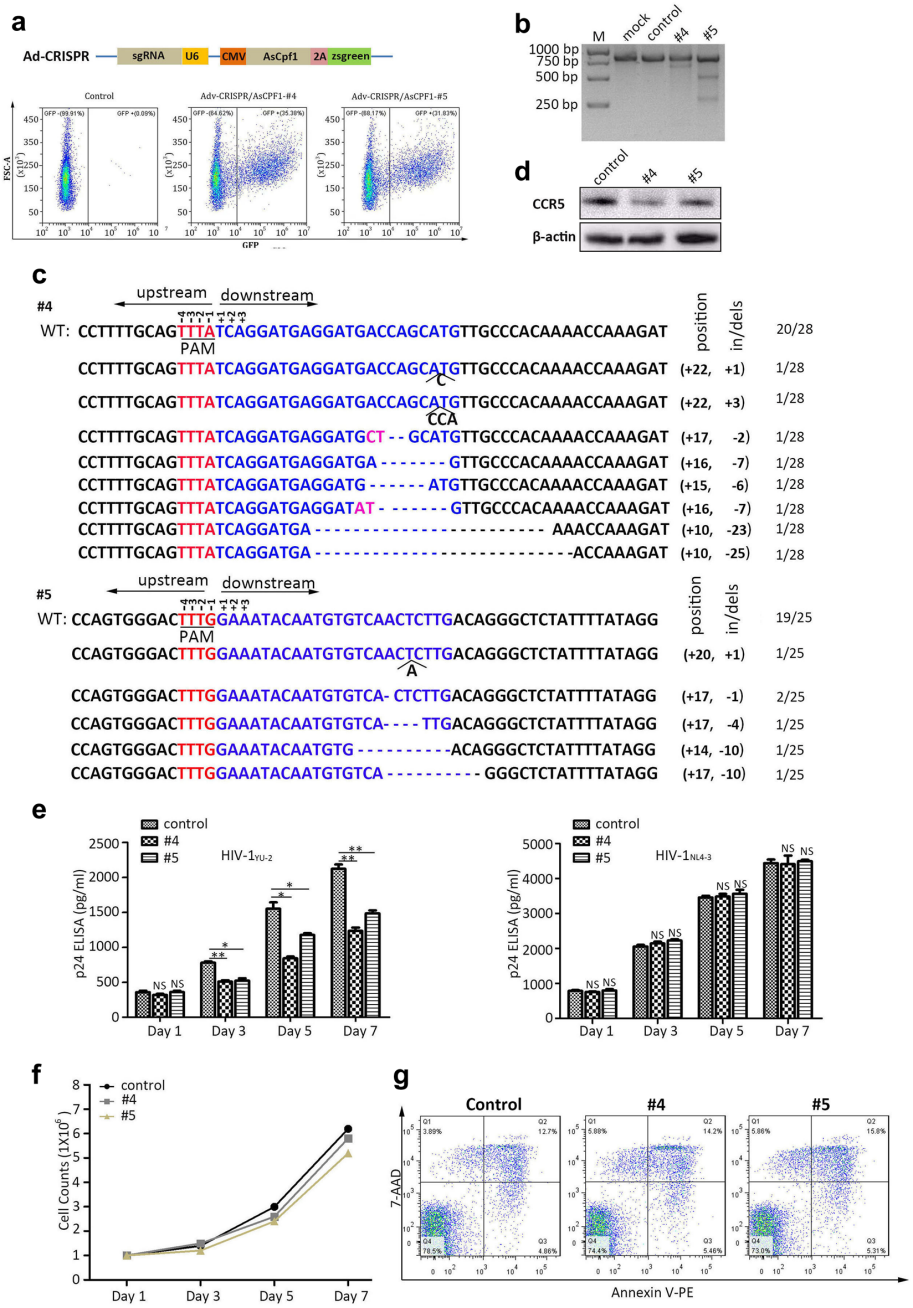
Adv-CRISPR/AsCpf1 mediated *CCR5* ablation suppresses HIV-1 infection in primary CD4^+^T cells. **a** The schematic diagram of the construction of CRISPR/AsCpf1 packaged adenovirus and corresponding transduction efficacy in primary CD4^+^T cells. **b** 300 ng of the CCR5 amplicons were identified with T7E1 after AdV-CRISPR/AsCpf1 transduction. **c** The CCR5 PCR products of the Adv-CRISPR/AsCpf1-#4/#5 transduced primary CD4^+^T cells were ligated with PGEM-T vector after poly A adding, and treated with DNA sequencing. **d** 10 μg of CCR5 protein of each group was assessed with western blotting. **e** R5-tropic HIV-1_YU-2_ or X4-tropic HIV-1_NL4-3_ was used to infect the adenovirus transduced primary CD4^+^T cells, the HIV-1 levels of each cells were determined from day 1 to day 7 post-infection. **f** Cell counts of each group were detected after the packaged adenovirus transduction for 7 days. **g** The apoptosis were analyzed with FCS at day 5 post-transduction. Necrotic cells (Annexin V-/7AAD +), necrotic or late apoptotic cells (Annexin V +/7AAD +); early apoptotic cells (Annexin V +/7AAD-); viable cells (Annexin V-/7AAD-). The data shown were the mean ± SD of three independent experiments. *P < 0.05; **P < 0.01; ***P < 0.001; NS, not significant; t test

After successfully editing *CCR5* in suspension CD4^+^ SupT1-R5 cells with AsCpf1- #4/#5 contained lentivirus, we attempted to disrupt *CCR5* in primary CD4^+^T cells with the packaged lentivirus. Unfortunately, we were not able to disrupt the *CCR5* obviously even with different MOI. Thus, we tried to package the AsCpf1-sgRNAs into adenovirus, the transduction efficacy of the adenovirus was more than 30% when compared with control at MOI = 100 (Fig. 6a). To evaluate the disruption efficacy of *CCR5* by the Adv-AsCpf1-#4/#5 in primary CD4^+^T cells, the T7E1 assay was performed after the cells were transduced with adenovirus (MOI = 100) at day 5 (Fig. 6b). The lower migrating fragments in #4 and #5 revealed that Adv-AsCpf1-#4/#5 could successfully edit the primary CD4^+^T cells. DNA sequencing for the *CCR5* amplicon was used further to analyze the indels (Fig. 6c). From the statistic, 8/28 of #4 (28.6%) and 6/25 of #5 (24.0%) were edited with different indels respectively, which indicated the Adv-AsCpf1-#4/#5 could disrupt *CCR5* in primary CD4^+^T cells efficiently. To test if the *CCR5* ablation in genome level resulted in protein level regulation, the immunoblotting showed the obvious down-regulation of CCR5 when compared with control (Fig. 6d). Since the *CCR5* gene of primary CD4^+^T cells could be edited by Adv-AsCpf1-#4/#5, like TZM.bl and SupT1-R5 cell line, we next examined whether the edited primary CD4^+^ T cells could defense HIV-1 infection. R5-tropic HIV-1_YU-2_ and X4-tropic HIV-1_NL4-3_ were utilized to infect the cells (Fig. 6e), the supernatants of each group were collected and measured from day 1 to day7 with p24 ELISA. The results suggested that the *CCR5* editing by Adv- AsCpf1-#4/#5 in primary CD4^+^T cells protected the cells from R5-tropic HIV-1_YU-2_ but not X4-tropic HIV-1_NL4-3_ infection compared with that of cells transduced with empty vector. Moreover, we further investigated whether the editing of *CCR5* inhibited proliferation or induced apoptosis of the primary CD4^+^T cells. The cell counts were monitored post-transduction from day 1 to day 7 and the apoptosis rates were analyzed for each group at day 5. The results displayed that no obvious proliferation and apoptosis change were detected in *CCR5* editing groups (#4, #5) when compared with control cells, which indicated that the *CCR5* disruption took no effect on general biological process of cells (Fig. 6f, g).

### Specific disruption of *CCR5* by CRISPR/AsCpf1 does not induce detectable off-targets effects

**Fig. 7:**
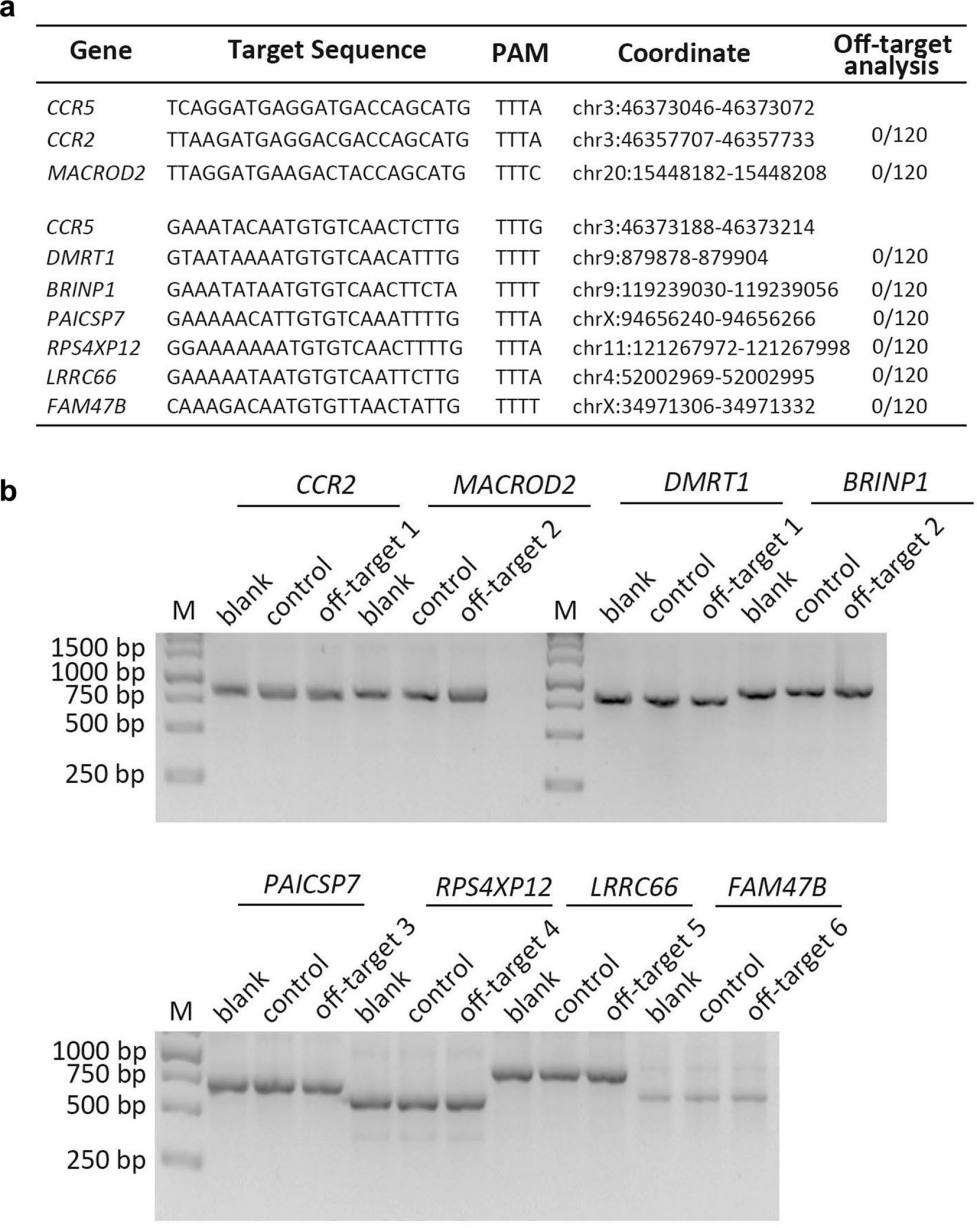
Specific disruption of CCR5 by CRISPR/AsCpf1 has no effect on detectable off-target. **a** The predicted off-target sites by online tool-CCTop were listed, and the possible indels of each amplicons were analyzed with DNA sequencing. **b** The T7 endonuclease 1 was further utilized to cut the products

While the CRISPR/AsCpf1 could mediate efficiently *CCR5* disruption in different cells, as the sgRNAs sequence of #4 and #5 we screened and identified may have some similarity with other locus in genome, it may result in inaccurate recognization by sgRNAs of #4 and #5 and cleavage by nuclease AsCpf1. Thus, the off-target analysis of #4 and #5 was performed and 2 off-target sites for #4 and 6 for #5 have been predicted with online tool CCTop (http://crispr.cos.uni-heidelberg.de/). All of the predicted sites were amplified by PCR with genome template from primary CD4^+^T cells. The amplicons were analyzed with T7E1 assay. As shown in (Fig. 7b), all of the sites exhibited no significant difference of fragments compared to control and blank. Meanwhile, DNA sequencing of all the sites were performed, while no cleavage of these sites were detected (Fig. 7a). The results indicated that the selected sgRNAs were high specific and safe, and no obvious off-targets could be observed. The related primers are listed in Additional file 1: Table S1.

## Discussion

In this study, we screened and identified two sgRNAs (#4, #5) targeting *CCR5* gene by CRISPR/AsCpf1. The CRISPR/AsCpf1-#4/#5 have been clarified that they could work efficiently to disrupt *CCR5* gene in different cell types of HIV-1 study (TZM.bl, SupT1-R5, primary CD4^+^T cells).The edited cells, compared with unedited control group showed that they could resist R5-tropic HIV-1 but not X4-tropic HIV-1 infection in all cell types. Meanwhile, we have verified that the *CCR5* edited SupT1-R5 cells took the selective advantage over unmodified control cells. Furthermore, the safety of the Adv-CRISPR/AsCpf1-#4/#5 mediated *CCR5* ablation in primary CD4^+^T cells also have been investigated and the disruption has no effect on cell proliferation and apoptosis, the off-target analysis of the two sgRNAs demonstrated that all the predicted sites showed no cleavage, which indicated that the disruption was relatively safe for gene therapy in the future.

From previous study, CCR5 is the major co-receptor of HIV-1 entering target cells at the early stage, many studies involving *CCR5* disruption have been reported by different gene editing tools, such as ZFN, TALEN and the newly developed CRISPR/Cas9. However, the ZFN and TALEN show many limitations, such as complex design, high off-target rate and not available to most of labs. Several studies associated with *CCR5* ablation by CRISPR/Cas9 have been reported recently, for example, Xiao et al. have reported that the CRISPR/SaCas9, a Cas9 version from *Staphylococcus aureus*, could efficiently edit the *CCR5* in different cell types including human CD34+ hematopoietic stem/progenitor cells, and the results in humanized mice have clarified that *CCR5* disruption via lenti-CRISPR/SaCas9 renders CD4^+^ T cells survival from HIV-1 infection [33]. A recent clinical study by Xu et al. via transplanting the *CCR5*-edited stem cells by CRISPR/SpCas9 to a patient with AIDS and leukemia, has been verified that the *CCR5* edited cells could resist HIV-1 infection, for a short time suspension of HAART promoted the editing efficacy. Meanwhile, the disruption efficacy in cells could be detected for 19 months without unspecific targeting and other side effects. Nevertheless, attempts should be made to study some other gene tools with much more easy design and higher safety. CRISPR/Cpf1 is a member of CRISPR/Cas family. Compared to the well known CRISPR/Cas9, the technology displays some differences, such as the recognization of target can be processed without the tracRNA, for the Cpf1 displays not only endonuclease activity but also exonuclease activity, which assists the mature of crRNA. The PAM sequence of CRISPR/Cpf1 is 5′-TTTN-3′- but not 5′-NGG-3′- in CRISPR/SpCas9 or 5′-NNGRRT-3′ in CRISPR/SaCas9, which makes the CRISPR/Cpf1 may take advantages over CRISPR/Cas9 in design and off-target concern. According to the studies of Benjamin et al. and Daesik et al., the endonuclease Cpf1 exhibits much more sensitivity to mismatches than spCas9, with no more than two single mismatches resulting in almost complete loss of Cpf1 activity at positions 1–18 (numbered 1–23 in the 5′ to 3′ direction). Furthermore, the two studies have also verified that the potential off-target sites of AsCpf1 were less than spCas9 [34, 35]. Interestingly, in predication of the off-target sites about the two sgRNA(#4, #5) using by CRISPR-AsCpf1 online, only few of the sites were presented (< 10), other than our previous work about CRISPR/Cas9 in off-target sites predication with tens to hundreds candidates. Besides, although the results of the top 3 off-target candidates analysis about spCas9-#1, #2 in our study exhibited no disruption, it should be considered that the sites we selected were less than 2% of all the predicted sites. So our results further indicated the superiority of CRISPR/AsCpf1 in gene editing, and proved that the sgRNAs (#4, #5) we selected are quite specific. Also, our study about *CCR*5 disruption by CRISPR/Cpf1 was the first report of CRISPR/Cpf1 editing of HIV-1 co-receptor, our results demonstrated the efficacy and specificity of the CRISPR-AsCpf1 sgRNAs (#4, #5). Moreover, the vector we used while investigating the efficacy of the system in primary CD4^+^T cells was adenovirus, the vector, not like lentiviral system integrating into genome that may induce cytotoxicity, was relatively safe and efficient.

It should also be noted in this study that CXCR4, as an another co-receptor of HIV-1, can function as the co-receptor for R5-tropic HIV-1 infection at late stage or some HIV-1 variants can enter cells via CXCR4 directly but not CCR5, just as the results of our study with additional experiments about *CCR5* and *CXCR4* editing simultaneously in TZM.bl resisting R5-tropic and X4-tropic HIV-1 infection, which makes the specific *CCR5* disruption only may not be sufficient. In addition, the CRISPR/AsCpf1-#4/#5 have not been investigated in vivo, such as in humanized mouse.

## Conclusions

In this study, we screened and identified two sgRNAs (#4, #5) for CRISPR/AsCpf1 editing *CCR5* in genome level with high specificity. We demonstrated that the CRISPR/AsCpf1-#4/#5 could ablate *CCR5* efficiently in different cell types (TZM.bl, SupT1-R5, primary CD4^+^T cells), and *CCR5* disruption protects the cells from R5-tropic HIV-1 but not X4-tropic HIV-1 without unspecific targeting and effects on cell proliferation and apoptosis. In addition, we clarified that the *CCR5* disrupted cells took selective advantages over unedited cells during R5-tropic HIV-1 infection. Our study suggests that *CCR5* disruption mediated by CRISPR/AsCpf1 can confer an effective and safe approach in HIV-1 gene therapy and may have potential of clinical application.

## Methods

### sgRNA design and lenti-CRISPR/AsCpf-sgRNA construction

The sgRNAs design for *CCR5* editing was based on online tool-CCTop (http://crispr.cos.uni-heidelberg.de/). 5 top ranking score of sgRNAs targeting *CCR5* exon were selected and synthesized with 5′-AGAT and 5′-AAAA overhangs. For the construction of lenti-CRISPR/AsCpf-sgRNA, the plasmid lenti-AsCpf1 (Addgene #PY108) was digested with endonuclease BsmB1 (Fermentas), the annealed sgRNAs were cloned into the digested lenti-AsCpf1 with T4 ligase.

### Cell lines culture and primary CD4^+^ T cell isolation

The studied TZM.bl and SupT1-R5 cells were cultured as previously described [20, 26, 33]. The primary CD4^+^T cells were isolated from healthy donors with Negative CD4^+^T Cells Isolation Kits (STEMCELL) according to the manufacturer’s instructions. The isolated primary CD4^+^ T cells were further cultured in CD3/CD28 coated 6-well plate with RPMI containing 10% FBS and 20 IU/ml recombinant human interleukin-2 (IL-2) was added.

### Virus (lentivirus, adenovirus) production and transduction

Lentivirus were produced with co-transfection of HEK293T cells with lenti-CRISPR/AsCpf1-#4/#5 or control, psPAX2, pMD2.G. After 60 h, the supernatants were collected and incubated with Lenti-X Concentrator (Takara), and the concentrated virus were further cryopreserved at − 80 °C.The target cells were transduced with concentrated lentivirus (MOI of 30) in the presence of 8 µg/ml polybrene (Sigma). The production of Adv-CRISPR/AsCpf1-#4/#5 or control were performed by HANBIO company (Shanghai, China). The Adv-AsCpf1 mediated transduction of primary CD4^+^T cells was according to instruction of manufacturer. Namely, 5 × 10^5^ cells/well in 12-well plate were infected with the adenovirus (MOI = 100) by spinning at 200*g* for 2 h, and the culture medium was replaced with fresh RMPI1640 after 12 h virus transduction. The transduced primary CD4^+^T cells were further cultured until downstream analysis.

### T7 endonuclease 1 (T7E1) cleavage assay and DNA sequencing

Genomic DNA of the treated cells were extracted with TIANamp Genomic DNA kit (TianGen) as the manufacturer’s instruction. The specific targets were amplified with PCR, and the amplicons were gel extracted. For the T7E1 assay, 300 ng of the amplicon was annealed and incubated with T7 endonuclease 1(NEB) for 30 min at 37  C. The cleavage was analyzed by 1.5% agarose gel electrophoresis. To further analyze the amplicon, the purified fragment was cloned into pGEM-T Easy vector (Promega), the detail of indels (insert/delete) were identified and compared with control.

### Luciferase activity assay and p24 gag detection by ELISA

The modified cells were infected with R5-tropic HIV-1_YU-2_or X4-tropic HIV-1_NL4-3_at MOI = 0.1 or 0.5, and the cells were further cultured after virus wash. For the detection of luciferase activity, the infected cells were washed and then lysed with 100 μl lysis buffer, and further measurement was performed with BrightGlo Luciferase assay according to the instruction of manufacturer (Promega). The supernatants of treated cells at indicated days post-infection were collected and the titer of virus were determined by p24 ELISA kit (ZeptoMetrix) as its instruction.

### Flow cytometry analysis

To assesses the disruption of CCR5 on the cell surface of TZM.bl, 5 µl FITC-conjugated mouse anti-human CCR5 antibody (Biolegend) were used and co-incubated with 5 × 10^5^ cells at the third day post-treatment for 15 min. The incubated cells were further washed for 3 times with 1× PBS, and then evaluated with CytoFLEX S (Beckman).The data were analyzed by CytExpert software. To evaluate the effects of adv-CRIPSR/AsCpf1 mediated *CCR5* disruption on the apoptosis of primary CD4^+^T cells, the apoptosis kit (US EverBright) was utilized. The primary CD4^+^T cells were incubated with 5 µl Annexin V-PE and 10 µl 7-AAD for 30 min, and the cells were analyzed with FCS as the instruction of manufacturer.

### Selective advantage analysis of CCR5-disrupted SupT1-R5 cells after R5-tropic HIV-1 challenge

To assess whether the *CCR5* edited cells were conferred with survival advantage over unedited control cells, the SupT1-R5 cells were transduced with associated lentivirus at MOI = 30. After 4 days post-transduction, the treated cells were infected with R5-tropic HIV-1_YU-2_ for 8 h. The cells were further cultured for 14 days after the infected cells were washed and replenished with fresh medium. The genomic DNA at day 0, 7, 14 post-infection were extracted and assessed with T7E1 assay. Consistently, the virus titer of HIV-1 were detected by p24 ELISA at day 1, 7 and 14.

### Off-target analysis

To evaluate the possible off-target of the selected sgRNAs (#4, #5), the sgRNAs were analyzed by online tool-CCTop (http://crispr.cos.uni-heidelberg.de/). According to the predicted results, all of the off-target sites for sgRNAs (#4, #5) were amplified with PCR and assessed with T7E1 assay. For further investigating the possible in/dels, the amplicons of treated cells were performed with DNA sequencing.

### Statistical analysis

Statistical analyses were performed using SPSS software, version 16.0. Differences between two groups were analyzed using the Unpaired Student’s t test. *p < 0.05; **p < 0.01, and ***p < 0.001 represents significant differences. NS represents not significant.

## Supplementary information

**Additional file 1: Table S1.** Primers used in this study. Primers for *CCR5*,*CXCR4* amplification and Cpf1-sgRNA-#4/#5 or Cas9-sgRNA-#1/#2 associated predicted off-target sites study.

**Additional file 2: Figure S1.** CCR5 editing by CRISPR/AsCpf1 and CRISPR/spCas9. A, target sequence of CRISPR/AsCpf1-#4 and CRISPR/spCas9-#1/#2. B, the cleavage efficacy of *CCR5* by CRISPR/AsCpf1-#4 and CRISPR/spCas9-#1/#2 at 72 h post-transfection in TZM.bl cells. C, DNA sequencing of the edited fragments. D, E, T7E1 and DNA sequencing analysis of the top 3 candidates of all the predicated off-target sites about spCas9-#1/#2.

**Additional file 3: Figure S2.** CCR5 and CXCR4 editing simultaneously by CRISPR/AsCpf1 protects the cells from CXCR4-tropic and CCR5-tropic HIV-1 infection. A, the target sequence of screened CRISPR/AsCpf1 usingCXCR4-sgRNAs. B, T7E1 confirmed editing efficacy of the screened CXCR4-sgRNAs. C, DNA sequencing of CXCR4 fragment after CRISPR/AsCpf1-CXCR4-#2 editing. D, the simultaneously ablation efficacy of CXCR4 and CCR5 after co-delivery of CRISPR/AsCpf1-CXCR4-#2 and CRISPR/AsCpf1-CCR5-#4 into TZM.bl cells. E, the CXCR4 and CCR5 modified TZM.bl cells or control were challenged with R5-tropic HIV-1_YU-2_ and X4-tropic HIV-1_NL4-3_ mix (1: 1) at MOI = 0.5. The data shown were the mean ± SD of three independent experiments. **P < 0.01; NS, not significant; Statistical analysis determined using unpaired t-test.

## Data Availability

All data generated or analyzed during this study are included in this published article.
