# Supplementary figures and images for: Genome editing of CCR5 by AsCpf1 renders CD4+T cells resistance to HIV-1 infection
Source: Cell Biosci. 2020 Jul 8;10:85. doi: 10.1186/s13578-020-00444-w (PMC7346486; doi:10.1186/s13578-020-00444-w)

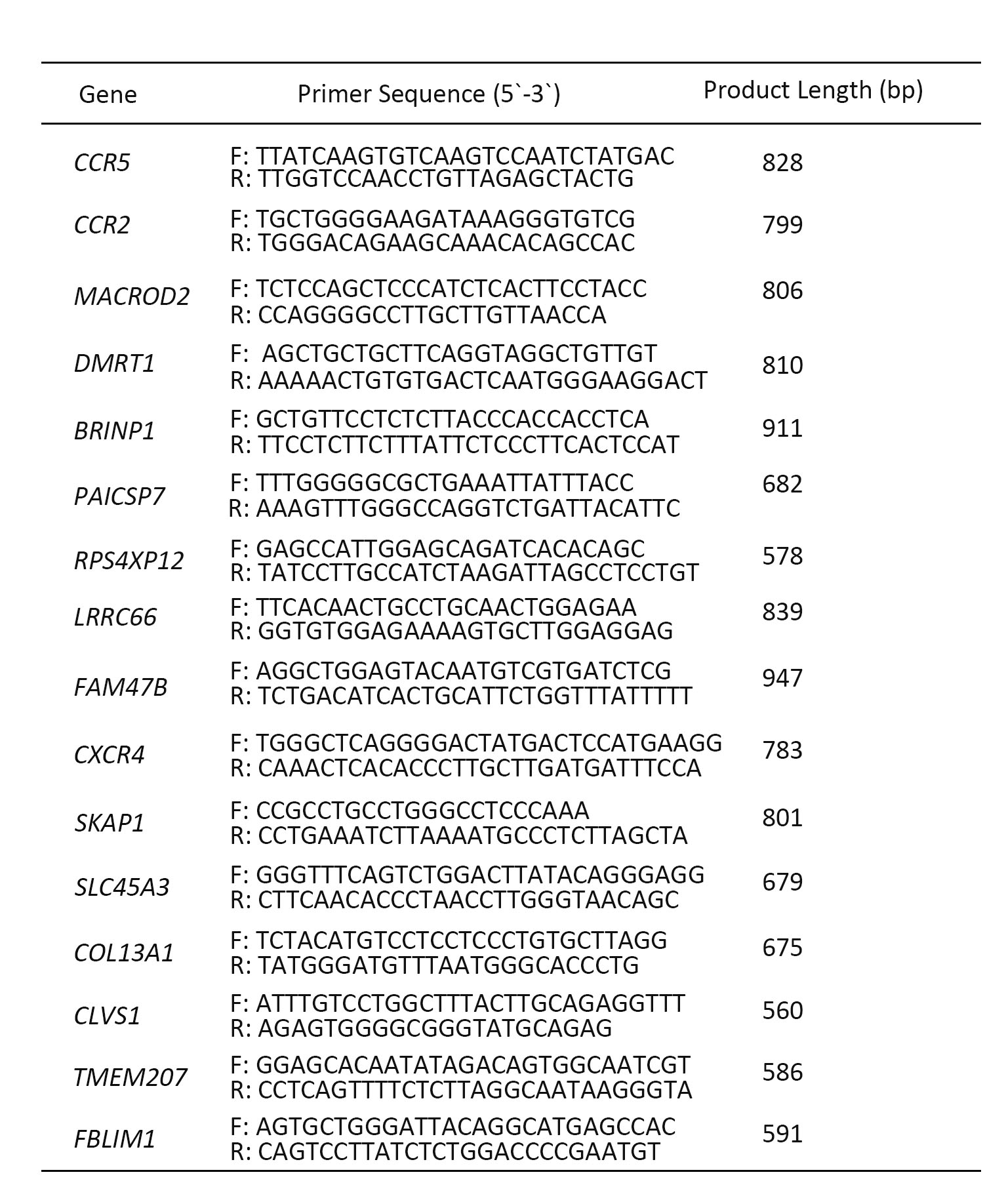

Supplement: Supplementary file 1 — Additional file 1: Table S1. Primers used in this study. Primers for CCR5,CXCR4 amplification and Cpf1-sgRNA-#4/#5 or Cas9-sgRNA-#1/#2 associated predicted off-target sites study. [file 13578_2020_444_MOESM1_ESM.jpg]

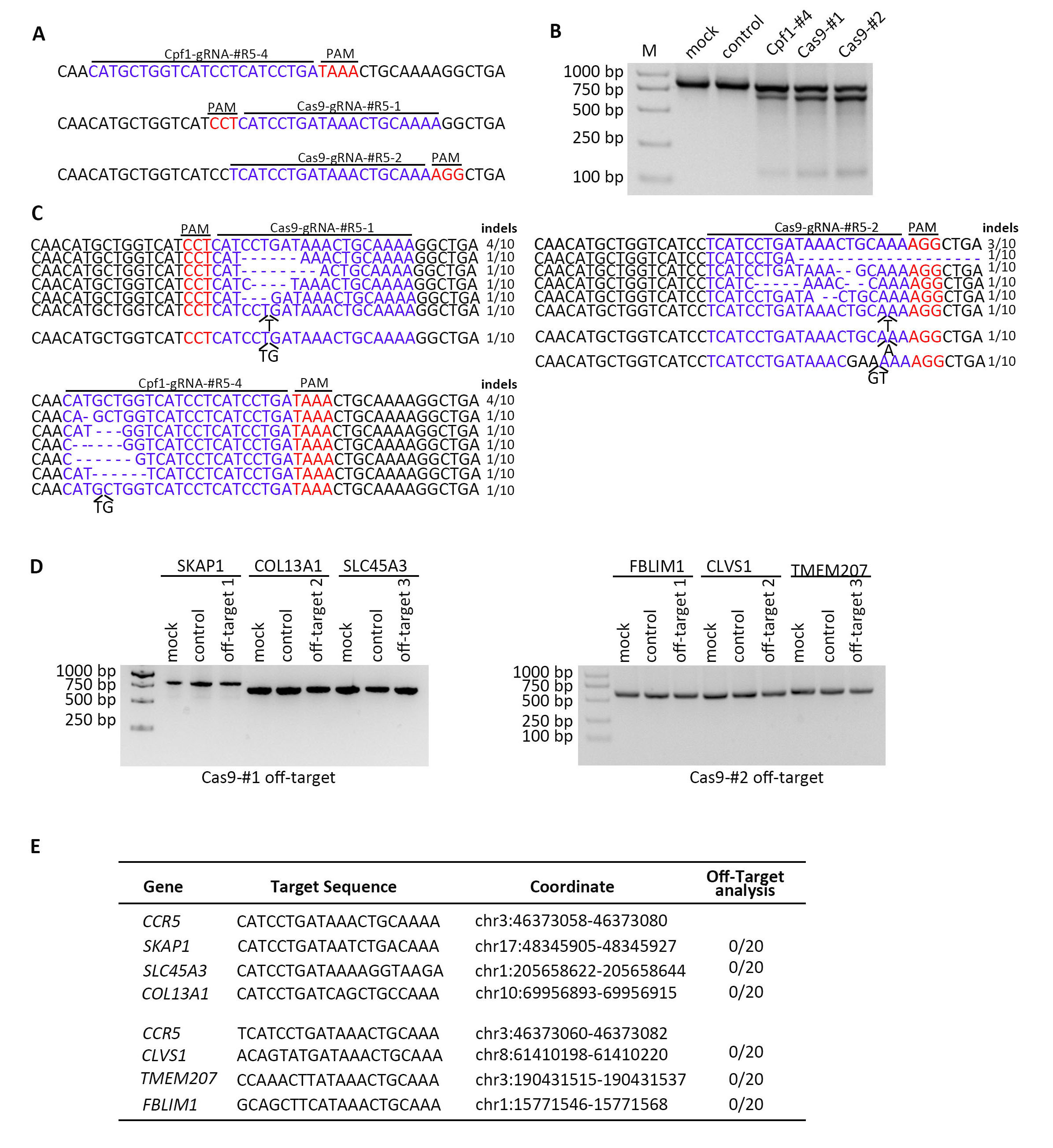

Supplement: Supplementary file 2 — Additional file 2: Figure S1. CCR5 editing by CRISPR/AsCpf1 and CRISPR/spCas9. A, target sequence of CRISPR/AsCpf1-#4 and CRISPR/spCas9-#1/#2. B, the cleavage efficacy of CCR5 by CRISPR/AsCpf1-#4 and CRISPR/spCas9-#1/#2 at 72 h post-transfection in TZM.bl cells. C, DNA sequencing of the edited fragments. D, E, T7E1 and DNA sequencing analysis of the top 3 candidates of all the predicated off-target sites about spCas9-#1/#2. [file 13578_2020_444_MOESM2_ESM.jpg]

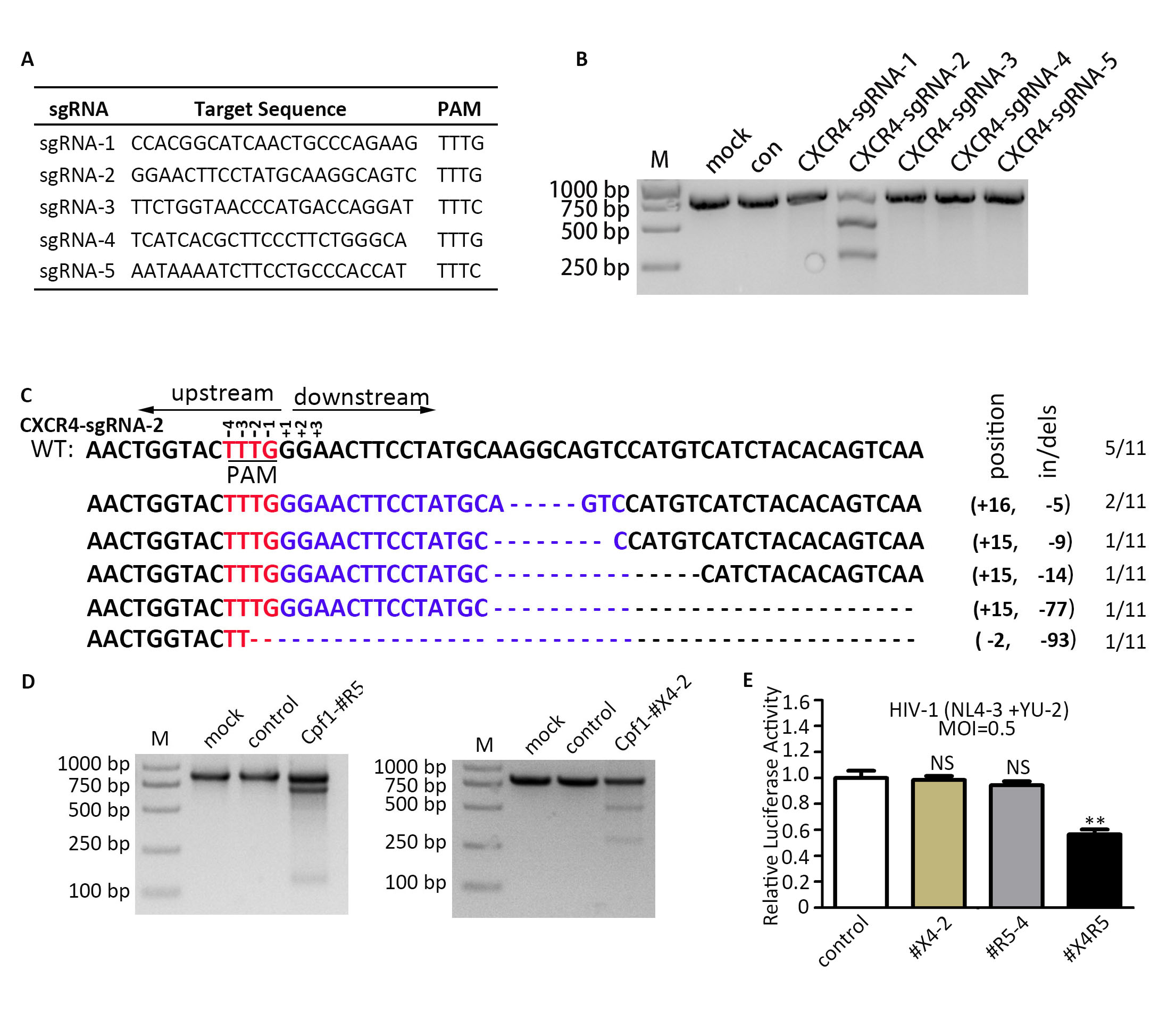

Supplement: Supplementary file 3 — Additional file 3: Figure S2. CCR5 and CXCR4 editing simultaneously by CRISPR/AsCpf1 protects the cells from CXCR4-tropic and CCR5-tropic HIV-1 infection. A, the target sequence of screened CRISPR/AsCpf1 usingCXCR4-sgRNAs. B, T7E1 confirmed editing efficacy of the screened CXCR4-sgRNAs. C, DNA sequencing of CXCR4 fragment after CRISPR/AsCpf1-CXCR4-#2 editing. D, the simultaneously ablation efficacy of CXCR4 and CCR5 after co-delivery of CRISPR/AsCpf1-CXCR4-#2 and CRISPR/AsCpf1-CCR5-#4 into TZM.bl cells. E, the CXCR4 and CCR5 modified TZM.bl cells or control were challenged with R5-tropic HIV-1YU-2 and X4-tropic HIV-1NL4-3 mix (1: 1) at MOI = 0.5. The data shown were the mean ± SD of three independent experiments. **P < 0.01; NS, not significant; Statistical analysis determined using unpaired t-test. [file 13578_2020_444_MOESM3_ESM.jpg]
